# Impacts of compounding drought and heatwave events on child mental health: insights from a spatial clustering analysis

**DOI:** 10.1007/s44192-023-00055-0

**Published:** 2024-01-02

**Authors:** Kelly Sewell, Sudeshna Paul, Kelley De Polt, Maggie M. Sugg, Ronald D. Leeper, Douglas Rao, Jennifer D. Runkle

**Affiliations:** 1 North Carolina Institute of Climate Studies, North Carolina State University, Raleigh, NC USA; 2 Nell Hodgson Woodruff School of Nursing, Emory University, 1520 Clifton Road, NE Atlanta, GA 30322-4027 USA; 3 Department of Biogeochemical Integration, Max Planck Institute for Biogeochemistry, Jena, Germany; 4 Department of Geography & Planning, Appalachian State University, Boone, NC USA

**Keywords:** Heatwave, Drought, Pediatric mental health, Suicide, Mood disorders, Compounding climate hazards

## Abstract

**Background:**

Concurrent heatwave and drought events may have larger health impacts than each event separately; however, no US-based studies have examined differential mental health impacts of compound drought and heatwave events in pediatric populations.

**Objective:**

To examine the spatial patterns of mood disorders and suicide-related emergency department (ED) visits in children during heatwave, drought, and compound heatwave and drought events. We tested whether the occurrence of compound heatwave and drought events have a synergistic (multiplicative) effect on the risk of mental health related outcomes in children as compared to the additive effect of each individual climate hazard. Lastly, we identified household and community-level determinants of geographic variability of high psychiatric burden.

**Methods:**

Daily counts of psychiatric ED visits in North Carolina from 2016 to 2019 (May to Sept) for pediatric populations were aggregated at the county scale. Bernoulli cluster analyses identified high-risk spatial clusters of psychiatric morbidity during heatwave, drought, or compound heatwave and drought periods. Multivariate adaptive regression models examined the individual importance of household and community-level determinants in predicting high-risk clustering of mood disorders or suicidality across the three climate threats.

**Results:**

Results showed significant spatial clustering of suicide and mood disorder risks in children during heatwave, drought, and compound event periods. Periods of drought were associated with the highest likelihood of spatial clustering for suicide and mood disorders, where the risk of an ED visit was 4.48 and 6.32 times higher, respectively, compared to non-drought periods. Compounding events were associated with a threefold increase in both suicide and mood disorder-related ED visits. Community and household vulnerability factors that most contributed to spatial clustering varied across climate hazards, but consistent determinants included residential segregation, green space availability, low English proficiency, overcrowding, no broadband access, no vehicle access, housing vacancy, and availability of housing units.

**Conclusion:**

Findings advance understanding on the locations of vulnerable pediatric populations who are disproportionately exposed to compounding climate stressors and identify community resilience factors to target in public health adaptation strategies.

**Supplementary Information:**

The online version contains supplementary material available at 10.1007/s44192-023-00055-0.

## Introduction

Climate change has already contributed to a rise in the occurrence of extreme events globally (NCA5). But the recent Sixth Assessment Report (AR6) of the Intergovernmental Panel on Climate Change (IPCC) has drawn attention to an understudied trend in the simultaneous occurrence of multiple climate hazards, like heatwaves and droughts, that co-occur in the same geographic region or close in time [1]. When multiple climate hazards or drivers co-occur —referred to as a compound event—the societal impacts of these simultaneous hazards are often amplified [2, 3]. The co-occurrence of these two extremes has resulted in excess heat-related deaths [4, 5], catastrophic forest fires in the summer [6–8], crop failure [9, 10], and widespread economic losses globally [11–14]. Climate change is causing a shift in increased warming that coincides with an increase in the intensity, frequency, duration, and seasonal length of drought events in North America [15]. The probability of co-occurring droughts and heatwaves has increased during the observational period in many areas of the world and will continue to increase, with projections showing a potentially doubling in occurrence for the northern hemisphere [15–20].

Recent examples of drought events with low precipitation and extreme temperature include the 2014 California droughts [13] as well as the 2018 and 2022 European Summers [21]. In the case of the 2014 California droughts, the precipitation was not at a record low value, though the co-occurring high temperatures created the conditions for the extreme event. Traditional risk assessment methods are based on only one climate hazard and may significantly underestimate the health impacts of climate extremes, particularly those co-occurring in time and space in US communities [22, 23].

Climate change will continue to increase the intensity and frequency of hot and dry extremes, including heatwaves and droughts [1, 24], with significant implications for child health [25]. In fact, recent evidence shows that under current high emission climate conditions, children born in 2020 are expected to encounter a two to seven fold increase in extreme weather, particularly heatwaves events [26]. The impacts of the climate crisis on child mental health is a growing area of concern, particularly when considering the potential impact these events may have on childrens’ rapidly developing brains [22, 23]. Emerging research suggests that climate-related extreme heat events have led to increased mental-health related emergency department (ED) visits, including self-inflicted injury/suicide and aggression among children [27, 28] and emotional distress and behavioral difficulties in adolescents [29]. Extreme temperatures have also been associated with an increase in emergency department visits for mood and behavioral disorders in children under the age of fourteen [30]. Population-based exposure to extreme temperatures and drought has been separately associated with deterioration in mental well-being and increased psychiatric-related ED visits, including elevated risks for suicide and violent behavior, particularly for adult populations with pre-existing mental and behavioral disorders [25, 30–35] but less is known about the health impacts of these co-occurring climate extremes on child and adolescent populations [36].

Children and adolescents may experience these impacts from extreme weather disproportionately due to their less developed regulatory systems and limited capacity to mitigate and/or adapt to the impacts of extreme climate events [37]. While the underlying mechanisms by which heat or drought alone, or heat and drought working synergistically, may increase the risk of mental health consequences in young people are unclear, potential biological pathways linking these two physical hazards to mental health may involve the following: dehydration or electrolyte imbalance [38, 39], altered brain activity and connectivity [40], medications used to treat certain conditions may reduce thermoregulatory capacity [40], sleep disruptions [40], or prior exposure to direct trauma from a heatwave or drought event [41]. More research is needed to understand the protective effect of social and environmental factors within a community that reduces the mental health consequences of extreme drought and heatwaves events in children.

The Southeastern United States region has historically experienced higher amounts of precipitation and water availability, but has been subject to multiple droughts in recent decades [42]. The U.S. National Climate Assessment (NCA) reports that extreme wetness and dryness are expected to increase across the U.S. by the end of the century, with the length of consecutive dry days to increase by 30% and longest consecutive dry period to increase by up to five days in the Southeastern U.S [43]. Temperature and precipitation have a strong negative correlation during the summer [15] due to weather systems that are favorable for extreme heat while unfavorable for rain [44].

To our knowledge, no US-based studies have examined differential mental health impacts of compounding drought and heatwave events in pediatric populations. This critical research gap is concerning due to the forecasted increase in concurrent heat and drought events as global warming levels increase [15–20, 42]. To address this gap, we examined the spatial patterns of child, adolescent, and young adult mental health-related emergency department (ED) visits during heatwaves, droughts, and compounding heatwave and drought events. Additionally, we tested whether the compounding nature of heatwave and drought events have a synergistic (multiplicative) effect on the risk of mental health related outcomes in children as compared to an additive effect of each individual climate hazard. We then examined the influence of individual sociodemographic and household or community-level contextual factors that explain geographic variability of heat and drought-related ED admissions among youth. Findings can advance understanding on the locations of vulnerable child and adolescent populations who are being disproportionately exposed to compounding climate stressors and identify community-level resilience pathways to target public health adaptation strategies that prioritize safeguards for child health.

## Methods

### Study population and time period

The study population included children, adolescents, and young adults aged 6–24 years who had a mental health-related visit to the emergency department (ED) during the warm season between the years 2016 and 2019 in North Carolina. For the purposes of this study, the warm season was defined as May to September. Daily ED visit data were obtained from the UNC Sheps Center and the study protocol was approved by the institutional review board (protocol 23452). Individual factors included in the ED visit record included participant age (0–12 years, 13–18 years, 19–24 years), race (White, Black/African American, Other), ethnicity (Hispanic, non-Hispanic), sex (male, female), and payor (commercial, Medicaid, Other government, uninsured).

### Child mental health outcomes

We relied on International Classification of Diseases, Tenth Revision (ICD-10) diagnosis codes to flag daily ED visits in each county for the following mental health outcomes: (1) suicide attempts (ICD-10: R45851) and (2) mood affective disorders (ICD-10: F30-F34, F38, F39) (i.e., a class of serious mental illnesses that encompass all types of depression and bipolar disorders).

### Compound hazard exposure assessment

We characterized county-level exposure to three separate climate stressors for the warm season between 2016 and 2019: (1) heatwave days, (2) drought days, and (3) compound heatwave and drought days.

#### Defining heatwaves days

**Fig. 1:**
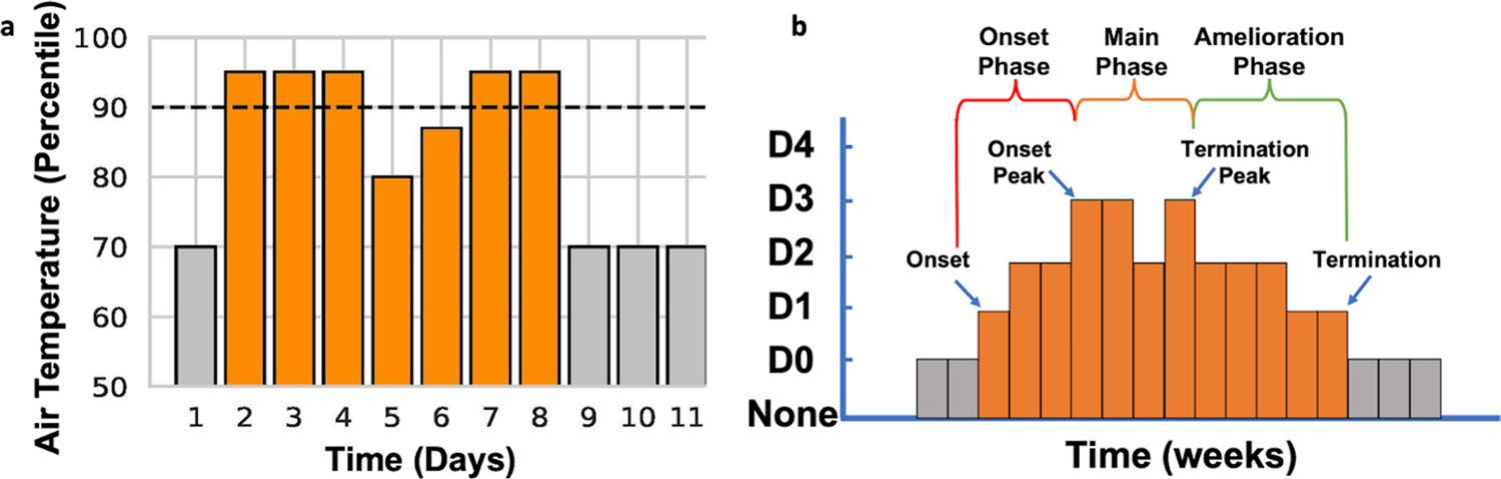
Heatwave (**a**) and drought (**b**) event definitions. **a** Heatwaves were defined as having at least 3-days above the seasonally adjusted 90th percentile ending the last day above the 90th with 3 consecutive days below the threshold. **b** Drought events were defined at a weekly scale when D1 (moderate drought) conditions were reached and ended after three consecutive weeks below Moderate (D1) drought conditions

Daily average air temperature was obtained for every county in North Carolina from 2000 to 2019 using NOAA’s nClimGrid dataset to establish the local climatology [45]. A heatwave event was defined to begin the first day the daily average temperature in a county exceeded the 90th percentile threshold for 3 consecutive days [46–51]. Similarly, a heatwave event ended the first day when temperature conditions dropped below the 90th percentile and remained below this threshold for three consecutive days. In order to capture early and late summer heatwave events, the 90th percentile was evaluated for each calendar day using a centered 31-day moving window (15 days on either side of date of interest) that spans over all years in the climatology data record for each county (2000 to 2019). For instance, the heatwave criteria for May 1st would be based on the 90th percentile of all daily temperature observations captured in the climatology of a particular county (2000 to 2019) between April 15th and May 15th [52]. (Fig. 1a).  For a sensitivity analysis examining heatwave definition, we also flagged high intensity heatwave days by relying on Nairn and Fawcett’s excess heat factor (EHF) metric for heatwave intensity (2015). EHF is unique in that the metric simultaneously takes into account (1) the local climatology of a particular county (i.e., identifies a heatwave day when 3 or more days that exceeds the 95th percentile of average temperature) and (2) the population acclimatization to temperature within the past 30 days. To derive severity, we then computed the 85th percentile of all positive EHF values within the climatology of a particular county and identified the heatwave as being high intensity.

#### Defining drought days

Weekly drought data for each county was obtained from the U.S. Drought Monitor (USDM). The USDM uses a convergence of evidence approach based on both a series of drought indicators (i.e., standardized precipitation index, Palmers Drought Severity Index, and Evaporative Drought Demand Index, among others) and drought reports from local experts (e.g.,, State Climatologists) to categorized drought into the following conditions: None, D0 (Abnormally Dry), D1 (Moderate drought), D2 (Severe drought) D3 (Extreme Drought) and D5 (Exceptional Drought) [53]. The first week in which a county was determined to be in drought if the drought category exceeded D1 (Fig. 1b) and the end of the last week in D1 conditions followed by three consecutive weeks of D0 or None similar to Leeper et al. [54] approach. If a designated week within a county was in drought conditions, every day of that week was flagged as a drought day.

#### Compound heatwave and drought days

Compound event days (yes/no) was defined as a day in which a heatwave and drought co-occurred in the same county. Compound event days were operationalized to represent multiple heatwaves within a single drought and were considered as separate events.

### Contextual community-level data

Decennial Census estimates for each county were used to calculate the Index of Concentration at the Extremes (ICE) [55], a metric that quantifies the extent to which a population is concentrated into extremes of racial segregation (i.e., majority Black compared to majority White communities), economic segregation (majority low-income compared to majority high-income communities), and the combined racialized economic segregation (i.e., majority Black, low-income compared to majority White, high-income) [56–58]. The ICE metric characterizes spatial representations of inequality and ranges in value between -1 to 1; where a value of -1 indicates all of the population is within the most economically or racially segregated or deprived group while a value of 1 means that all of the population is within the most privileged group [55]. This measure has been used in other climate impact papers on population health [59].

Data on individuals and households within each NC county were obtained from the Community Resilience Estimates Equity Supplement (CRE for Equity) using the 2019 American Community Survey (ACS) 1-year microdata [60]. Variables included were known to influence vulnerability, exposure and adaptive responses to climate hazards, including households with low educational attainment, no vehicle, low occupancy rate, overcrowding, high proportion of children and aging populations (over 65), low English proficiency, no health care coverage, and no broadband access [59, 60] (See Supplemental Table 1). Lastly, total greenspace per person in each county was derived using data from the USGS PADUS [61] and ParkServe [62] using the methodology outlined in Runkle et al. [63]. Earlier studies have shown the protective child mental health benefits of high availability of residential green and park space [64].

### Statistical analysis

#### Spatial clustering approach

For this retrospective ecologic study, a spatial scan approach employing the Bernoulli model within the SaTScan version v10.0.2 software was used to examine the purely spatial clustering of mental health-related ED visits among youth inside and outside heatwave, drought, and compounding heatwave and drought events. For the example of heatwaves, we compared the binary occurrence of suicide-related ED visits during a heatwave event (i.e., cases) compared to a non-heatwave event (i.e., controls) aggregated to the county level for the summer season. An elliptical moving window of varying diameter was used to better estimate the number of observed and expected cases for each county [65], with a size that included up to 25% of the total cases. We performed a sensitivity analysis using varying windows up to 50%; however, because the spatial scan is at the county scale, a larger geographic area, we opted for a smaller window to allow for the identification of more local clusters [66]. The window with the maximum likelihood is typically the cluster least likely to have occurred by chance. The maximum likelihood ratio test with the Monte Carlo method using 999 permutations was used to test the alternative hypothesis that there was an elevated risk within the window compared to outside the window. Clusters with a p < 0.05 were considered to be statistically significant, and the cluster with the highest LLR was considered to be the most likely or the primary cluster [67]. Counties within statistically significant primary and secondary clusters (RR > 1.0) who had a local cluster in which RR < 1.0 were removed to exclude neighboring regions with non-elevated risk [68]. We then matched individual-level ED admissions with whether or not the case occurred in a county designated as a high versus low risk cluster for each of the climate hazards.

#### Assessing additive, multiplicative, and synergistic interaction

To better assess the public health relevance of intervening on individual versus joint effects of each climate threat, interaction on the additive and multiplicative scale was assessed [69]. We estimated the relative excess risk due to interaction (RERI) to approximate additive interaction using risk ratios in lieu of risks [70]. The synergy index (S) was also approximated as an alternative measure of additive interaction [71] to determine if the risk ratios for both exposures (i.e., heatwave and drought events) exceeded a value of one and whether or not this exceedance was larger than the rate ratio for the two separate climate threats that each exceeded one [69, 72].

#### Household and community determinants of geographic variability of mental health risks

Next, we used multivariate adaptive regression splines (MARS), a popular machine learning method, to model the relationships between individual, household, and community-level predictors and the frequency of each outcome for the three distinct climate threats in the high-risk clusters. Because of the likely non-linear and complex nature of these relationships, we preferred using the MARS algorithm over traditional regression analysis to express the predictor-outcome relationships as piecewise linear models by assessing cutpoints (knots). One advantage of the MARS models is that they can account for nonlinear relationships between the predictor and response variables. Furthermore, the exact form of the nonlinearity does not need to be known or specified explicitly prior to model training; the estimation algorithm will search for and discover nonlinearities in the data that help maximize predictive accuracy. This method is also useful when many variables are considered and allows for the rapid interrogation of a large number of regressions that search all possible combinations of predictors [73, 74]. In our case, the univariate counts for each outcome in the high-risk clusters were modeled using a Poisson distribution and forward and backwards selection methods were used to identify the predictors retained in the final model [75, 76]. We then examined the individual importance of each household or community-level factor in predicting high-risk clusters for pediatric suicide or mood disorders separately for each climate hazard. Variable importance in this non parametric analysis can be conceptualized as the explained variability in the outcome of interest (i.e., mood disorder or suicide-related ED admission) relative to the top-ranked predictors. A sensitivity analysis was performed to examine the influence of heatwave definition. Our alternative definition for heatwave included the high intensity heatwave metric and all MARS models were performed examining high intensity heatwave and compound high intensity heatwave and drought events for each outcome separately. Multivariate adaptive regression models were performed using the Adaptivereg procedure in SAS 9.4 [77, 78]. Data were first partitioned into training, testing, and validation using a third of the data. The generalized cross validation (GCV) and the GCV R-square criterion value were used to determine model fit [77, 79].

## Results

**Fig. 2:**
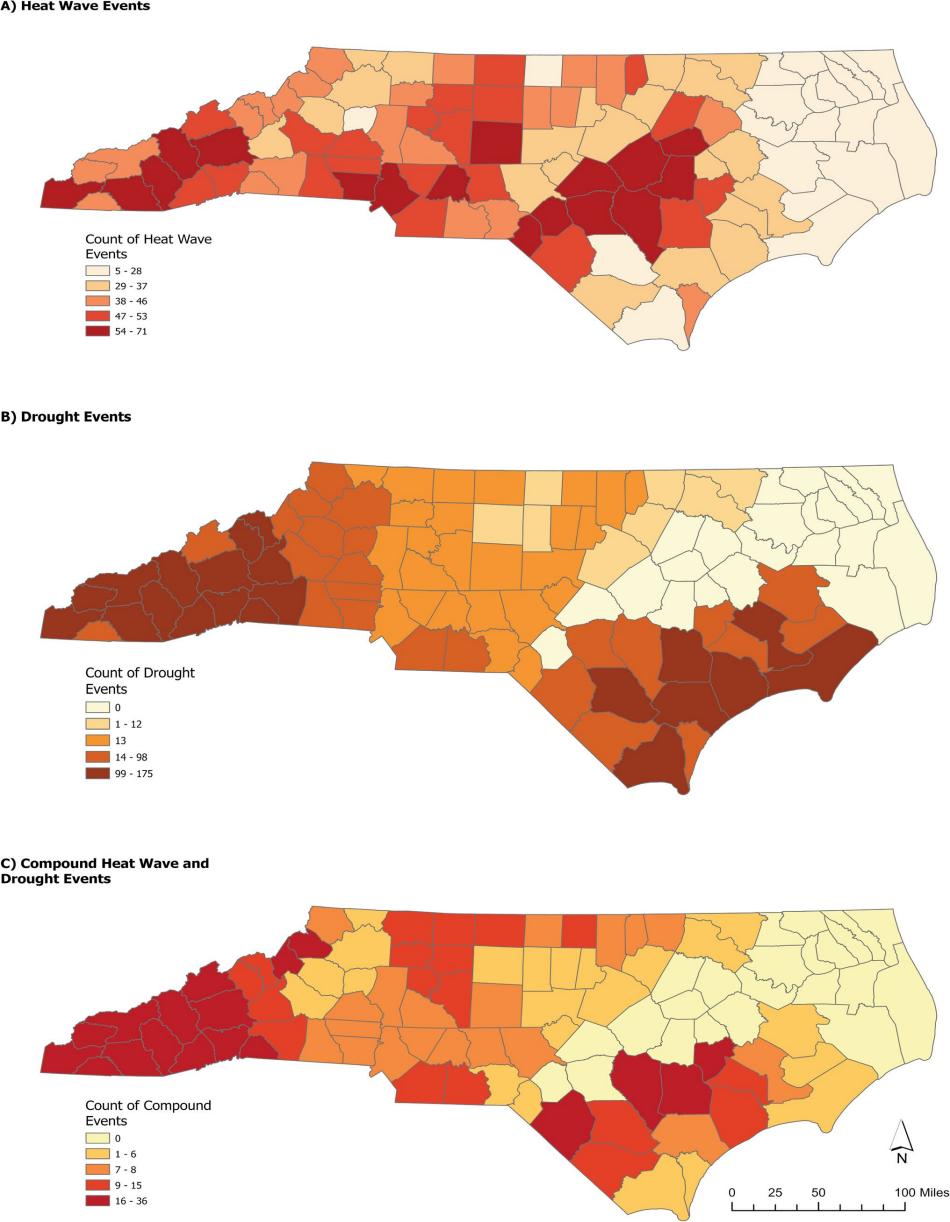
Total counts of **a** heatwave, **b** drought, and **c** compound heatwave and drought events by county in North Carolina, 2016–2019

Table 1 shows the demographic characteristics of children presenting to the ED with a mood- or suicide-related complaint in high-risk spatial clusters for each climate threat. In general, a higher proportion of African American (AA) youth presented to the ED with a mood disorder during a heatwave and a higher proportion of cases with a mood disorder during a drought event were female, younger than 12 years of age, identified as AA, and were insured by Medicaid. A higher number of heatwaves occurred in the western portion of the state and droughts in the central part where the Piedmont region transitions to the Coastal Plains; compound events clustered in the Southern Mountains and in central Coastal Plain (Fig. 2). Temperatures were lower during compounding heatwave and drought days compared to heatwave days alone (Supplemental Table 2), but results are sensitive to location (e.g., elevation, coast) and time of the year (e.g., early or late warm season).

**Table 1:** Demographic characteristics of pediatric populations presenting to the ED for mood disorders and suicidality in high- versus low-risk clusters, North Carolina (May to September 2016–2019)

	Mood Disorders						Suicidality					
	Heatwave		Drought		Compound		Heatwave		Drought		Compound	
	High-risk cluster		High-risk cluster		High-risk cluster		High-risk cluster		High-risk cluster		High-risk cluster	
	Yes	No	Yes	No	Yes	No	Yes	No	Yes	No	Yes	No
	n (%)	n (%)	n (%)	n (%)	n (%)	n (%)	n (%)	n (%)	n (%)	n (%)	n (%)	n (%)
Age in years												
0–12	135 (5.12)	129 (4.89)	110 (6.20)	65 (3.66)	21 (4.53)	29 (6.25)	72 (5.35)	123 (9.14)	71 (8.23)	47 (5.45)	16 (6.06)	18 (6.82)
12–18	816 (30.93)	1095 (41.51)	419 (23.61)	820 (46.20)	113 (24.35)	221 (47.63)	403 (29.96)	591 (43.94)	428 (49.59)	219 (25.38)	107 (40.53)	95 (35.98)
19–24	171 (6.48)	292 (11.07)	249 (14.03)	112 (6.31)	33 (7.11)	47 (10.13)	54 (4.01)	102 (7.58)	69 (8.00)	29 (3.36)	15 (5.68)	13 (4.92)
Total	1122 (42.53)	1516 (57.47)	778 (43.83)	997 (56.17)	167 (35.99)	297 (64.01)	529 (39.33)	816 (60.67)	568 (62.42)	342 (37.58)	138 (52.27)	126 (47.73)
Race												
White	574 (22.40)	1023 (39.93)	859 (49.71)	377 (21.82)	133 (30.02)	167 (37.70)	264 (20.31)	522 (40.15)	411 (49.16)	164 (19.62)	97 (38.19)	64 (25.20)
Black/African American	392 (15.30)	341 (13.31)	191 (11.05)	149 (8.62)	20 (4.51)	90 (20.32)	182 (14.00)	202 (15.54)	76 (9.09)	90 (10.77)	27 (10.63)	36 (14.17)
Other	123 (4.80)	109 (4.25)	108 (6.25)	44 (2.55)	10 (2.26)	23 (5.19)	54 (4.15)	76 (5.85)	66 (7.89)	29 (3.47)	9 (3.54)	21 (8.27)
Total	1089 (42.51)	1473 (57.49)	1158 (67.01)	570 (32.99)	163 (36.79)	280 (63.21)	500 (38.46)	800 (61.54)	553 (66.15)	283 (33.85)	133 (52.36)	121 (47.64)
Ethnicity												
Non-Hispanic	1001 (39.39)	1326 (52.18)	978 (60.52)	529 (32.74)	136 (31.05)	261 (59.59)	450 (34.88)	718 (55.66)	461 (59.18)	256 (32.86)	110 (44.53)	111 (44.94)
Hispanic	90 (3.54)	124 (4.88)	61 (3.77)	48 (2.97)	14 (3.20)	27 (6.16)	63 (4.88)	59 (4.57)	31 (3.98)	31 (3.98)	12 (4.86)	14 (5.67)
Total	1091 (42.94)	1450 (57.06)	1039 (64.29)	577 (35.71)	150 (34.25)	288 (65.75)	513 (39.77)	777 (60.23)	492 (63.16)	287 (36.84)	122 (49.39)	125 (50.61)
Sex												
Male	434 (16.46)	531 (20.14)	441 (24.86)	218 (12.29)	69 (14.90)	102 (22.03)	221 (16.44)	329 (24.48)	227 (26.33)	101 (11.72)	60 (22.81)	38 (14.45)
Female	688 (26.09)	984 (37.32)	737 (41.54)	378 (21.31)	97 (20.95)	195 (42.12)	308 (22.92)	486 (36.16)	340 (39.44)	194 (22.51)	77 (29.28)	88 (33.46)
Total	1122 (42.55)	1515 (57.45)	1178 (66.40)	596 (33.60)	166 (35.85)	297 (64.15)	529 (39.36)	815 (60.64)	567 (65.78)	295 (34.22)	137 (52.09)	126 (47.91)
Payer												
Commercial	312 (11.84)	485 (18.41)	313 (17.63)	188 (10.59)	58 (12.50)	91 (19.61)	169 (12.59)	282 (21.01)	160 (18.54)	110 (12.75)	51 (19.32)	43 (16.29)
Medicaid	715 (27.13)	937 (35.56)	796 (44.85)	365 (20.56)	96 (20.69)	190 (40.95)	323 (24.07)	459 (34.20)	370 (42.87)	163 (18.89)	78 (29.55)	71 (26.89)
Other governmental	51 (1.94)	31 (1.18)	28 (1.58)	24 (1.35)	4 (0.86)	8 (1.72)	18 (1.34)	35 (2.61)	16 (1.85)	10 (1.16)	3 (1.14)	4 (1.52)
Uninsured	42 (1.59)	62 (2.35)	42 (2.37)	19 (1.07)	9 (1.94)	8 (1.72)	19 (1.42)	37 (2.76)	22 (2.55)	12 (1.39)	6 (2.27)	8 (3.03)
Total	1120 (42.50)	1515 (57.50)	1179 (66.42)	596 (33.58)	167 (35.99)	297 (64.01)	529 (39.42)	813 (60.58)	568 (65.82)	295 (34.18)	138 (52.27)	126 (47.73)

### Spatial patterns of psychiatric morbidity during heatwaves, droughts, and compound events

**Fig. 3:**
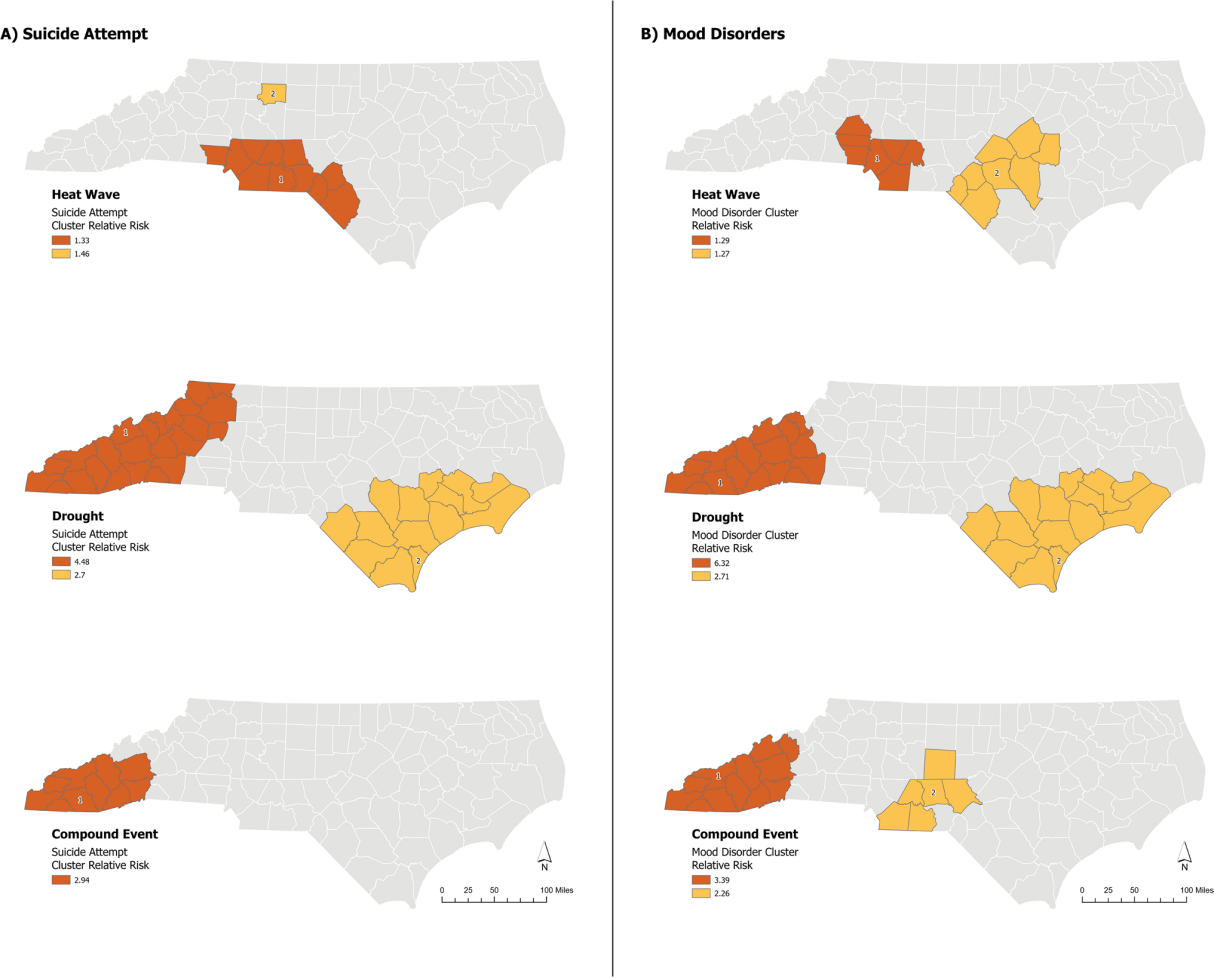
High-risk clusters for **A** suicide attempts and **B** mood disorders in pediatric populations for heatwave, drought, and compound heatwave and drought events in North Carolina, 2016–2019

Results revealed statistically significant spatial clustering for ED-related mood disorder and suicide visits, indicating that risks for these conditions were not randomly distributed across the three climate hazard types (Table 2, Fig. 3). The risk of a suicide-related admission during a heatwave was 1.33 times the risk compared to a non-heatwave period in the primary cluster and occurred in the metropolitan statistical area around Charlotte, the most populous city in NC. Pediatric mood disorder admissions were 29% higher during a heatwave compared to non-heatwave period; the primary cluster was also primarily located in Charlotte and then in Lumberton and Fayetteville, an area characterized by high heat. During drought events, we observed that visits for suicide and mood disorders in youth were 4.48 and 6.32 times higher, respectively, compared to non-drought periods in cluster locations located in the mountainous western portion of the state and along the Coastal Plain. Finally, a threefold increase in suicide and mood disorder-related ED visits in the primary cluster was detected during compound compared to non-compounding heatwave and drought days in the Southern Mountains.

**Table 2:** Summary statistics for suicide and mood disorder clusters in children during heatwave, drought, and compound heatwave + drought days compared to non-event days

	Cases	LLR	No. of counties	p-value	RR
Mood disorders					
Heatwave					
1st cluster	757	18.43	7	< 0.0001	1.29
2nd cluster	365	9.42	8	0.011	1.27
Drought					
1st cluster	671	616.57	16	< 0.0001	6.32
2nd cluster	448	149.4	14	< 0.0001	2.71
Concurrent					
1st cluster	92	43.34	12	< 0.0001	3.39
2nd cluster	49	12.01	6	0.0012	2.26
Suicidality					
Heatwave					
1st cluster	384	11.74	11	0.00263	1.33
2nd cluster	114	7.58	1	0.043	1.46
Drought					
1st cluster	324	205.49	24	< 0.0001	4.48
2nd cluster	204	68.79	14	< 0.0001	2.7
Concurrent					
1st cluster	49	18.84	10	< 0.0001	2.94
2nd cluster	88	5.2	12	0.351	1.54

*RR* Relative Risk, *LLR* Log likelihood ratio, *No.* number

### Additive versus multiplicative interaction

The joint effects of co-occurring exposure to both climate threats were not larger than the individual effects of exposure to either a heatwave or droughts event as revealed by negative interaction on both the additive and multiplicative scale (Table 3). Further, negative interaction for both outcomes demonstrated that the public health consequences of an intervention focused on reducing child ED visits for either suicide or mood disorders would be more effective if each hazard was targeted separately.

**Table 3:** Measures of interaction on additive and multiplicative scale for the primary cluster

	Measure	Equation	Mood disorders	Suicidality	
Additive interaction	RERIRR	RR_11_-RR_10_-RR_01_ + 1	−3.22	−1.87	< 0 = negative additive interaction or less than additivity
	Synergy	RR_11_−1/(RR_10_−1) + (RR_01_−1)	0.43	0.51	< 1 = negative additive interaction or less than additivity
Multiplicative interaction		RR_11_ / (RR_10_*RR_01_)	0.42	0.49	< 1 = negative multiplicative interaction or less than additivity

*RERI* Relative excess risk due to interaction, *RR* Relative Risk

### Contextual determinants of spatial patterns of psychiatric morbidity across climate stressors

For mood disorders, significant heterogeneity was observed concerning which household or community determinants contributed the most to high-risk geographic clustering across hazard types (Table 4). Factors like a higher proportion of older adults, availability of greenspace per person, higher rental/housing vacancy, residence in a racially segregated community, households characterized by overcrowding and a large proportion of Hispanic or Latino dwellers were the most predictive of high-risk clustering of mood disorders attributable to heatwaves. Factors that predicted living in a high-risk cluster for mood disorders during a drought event included housing availability, low educational attainment, lack of health care coverage, older age, low English proficiency, overcrowding, no vehicle access, and limited availability of greenspace. Finally, housing vacancy and available housing units, a higher proportion of veterans, households without a vehicle, residential segregation, older age, and greenspace per person explained about 94% of the total variance of high risk clusters for mood disorder during a compound event (i.e., GCV R-Square = 0.939). Social determinants like age, household overcrowding, low English proficiency and environmental determinants like availability of greenspace were shared vulnerability factors that contributed differentially to high-risk clustering of mood-related ED visits across the three climate hazards.

**Table 4:** Variable Importance identified in Adaptive regression for (A) Mood Disorders and (B) Suicidality

Residence in high-risk cluster during heatwave			Residence in high-risk cluster during drought			Residence in high-risk cluster during compounding drought and heatwave		
Variable	Bases	Importance		Bases	Importance		Bases	Importance
**(**A) **Mood disorders**								
% of population 65 years +	2	100	Estimated number of housing units	2	100	Homeowner vacancy rate	2	100
% total greenspace per person	2	78.59	% of population 25 years + that are HS graduates are higher	2	56.99	Number of housing units	2	97.16
% of population 18 years + that are civilian veterans	4	66.99	% of civilian noninstitutionalized population without health coverage	4	56.95	% of population 18 years + that are civilian veterans	4	90.52
Rental vacancy rate	2	59.94	% of population 65 years +	2	32.06	% of occupied housing units with 1.51 occupants/room	3	74.73
Homeowner vacancy rate	2	59.29	% of population Hispanic or Latino	2	14.74	% of occupied housing units with no vehicle	2	49.99
Residential segregation	2	54.49	% of occupied housing units with 1.51 occupants/room	2	13.01	Residential segregation	1	37.05
% of occupied housing units with 1.51 occupants/room	2	35.55	% of occupied housing units with no vehicle	2	10.59	% of population 65 years +	2	16.72
% of population Hispanic or Latino	2	29.97	% total greenspace per person	2	1.32	% total greenspace per person	1	5.59
Model fit statistics								
GCV		0.00842			0.14053			0.04506
GCV R-Square		0.98843			0.74174			0.93901
Effective degrees of freedom		39			39			37
Log likelihood		−1132.7834			−1298.2959			−175.85302
Deviance		21.5668			238.59184			17.70604
(B) **Suicidality**								
% of occupied housing units with 1.51 occupants/room	4	100	% of population 25 years + that are HS graduates are higher	2	100	% total greenspace per person	2	100
% total greenspace per person	3	98.98	Estimated number of housing units	2	96.43	Residential segregation	2	84.67
% of population Hispanic or Latino	4	93.05	% of population 65 years +	4	89.41	% of households with broadband internet	5	65.74
Residential segregation	5	78.68	Residential segregation	2	56.18	estimated number of Housing units	2	47.74
% of population 25 years + that are HS graduates are higher	2	34.16	% of occupied housing units with 1.51 occupants/room	4	42.14	% of occupied housing units with 1.51 occupants/room	2	44.32
			% of civilian noninstitutionalized population without health coverage	2	33.54	Rental vacancy rate	2	43.99
			% of occupied housing units with no vehicle	3	31.31	% of civilian noninstitutionalized population without health coverage	2	20.09
			% of households with broadband internet	1	23.85			
			% of population 18 years + that are civilian veterans	2	16.23			
Model fit statistics								
GCV		0.04761			0.14486			0.039
GCV R-Square		0.93524			0.73752			0.943
Effective degrees of freedom		39			45			37
Log likelihood		−559.1884			−624.15817			−141.789
Deviance		60.3768			112.31635			7.579

Youth at high risk for a suicide-related ED visit during a heatwave were more likely to live in communities characterized by overcrowding, poor availability of greenspace, low English proficiency, residential segregation, and low educational attainment compared to children in low-risk areas (Table 4). Geographic hotspots of suicide risk during drought events were similar in composition to geographic clusters for mood disorders with the exception that residential segregation, household access to broadband, and a higher proportion of veterans were more predictive of clustering. Unlike mood disorders, greenspace didn’t contribute to geographic variability of clustering of suicide risk on drought days. During compound extreme heatwave and drought days, greenspace availability, residential segregation, broadband access, housing availability, and access to health care coverage were the most predictive factors of geographic clustering of high suicide risks for pediatric populations compared to non-compound heatwave and drought days.

Results from the sensitivity analysis using the alternative heatwave definition did differ from the main analysis (Supp Table 3 and 4). For example, the sensitivity analysis examining high intensity heatwaves and mood disorders revealed a smaller list of predictive community-level factors and for factors that were present in both models, the order of importance varied. In general, models that did not use the high intensity heatwave definition performed better, indicated by a lower Log Likelihood ratio test in SatScan clusters and higher GCV R-Square in MARS models. This may be due to the fact that high intensity heatwaves are more severe and typically more rare occurrences; therefore, the sensitivity analysis were characterized by a smaller sample of cases.

## Discussion

This is the first US-based study to examine the mental health impacts of compounding drought and heatwave events on pediatric populations. Findings revealed significant geographic clustering of elevated risk for suicide and mood disorders in young people during heatwave, drought, and compound drought and heatwave days compared to non-event days. Periods of drought were associated with the highest risk of spatial clustering for mood disorders followed by suicide. Temporally compounding events were associated with a threefold risk in psychiatric admissions. Contrary to expected, results demonstrated that the combined effect of drought and heatwave days was less than the sum of the mental health effects for individual heatwave and drought events. Community and household vulnerability factors that contributed the most to geographic clustering of increased mood and suicide-related risks varied across the climate hazards. But consistent and shared spatial determinants of high-risk clustering of child psychiatric morbidity for all three climate stressors included residential segregation, green space availability, low English proficiency, overcrowding, no broadband access, no vehicle access, housing vacancy rates and availability of housing units. Local communities might target enhancing availability of more and greener park spaces, transportation and affordable housing access, as well as improving access to broadband internet in local climate policy and adaptation efforts.

Consistent with our findings, earlier studies have demonstrated elevated rates of mood disorders, like depression, in children during periods of prolonged drought [29, 80, 81]. Studies have also found a direct association between prolonged drought and worsened mental health in young people, particularly increased levels of emotional distress [29, 82] and elevated risk of suicide [83]. Constant and long term drought patterns have also been associated with an accumulated mental health burden for people living in rural areas in Australia, indicating that specific drought patterns may be linked to increased mental distress attributed to family concerns, financial stress, worry about the future brought on by a changing climate, social isolation, and perceived loss of connection to the land in agricultural communities [29, 84]. ED visits in adolescents for suicide and depression have also been shown to peak in the warm season [27, 85, 86], but not in all studies [30, 87].

While all children are at risk, our results showed that a higher physical hazard-health burden is shouldered by those in socially and economically overburdened communities and non-English-speaking households. Health risks from heatwaves have been shown to be greater in low-income communities of color, where discriminatory policies such as redlining have resulted in urban heat islands and fewer resources to protect children from heat [25]. Emerging research shows that the health impacts of climate-related extreme weather events have been more severe among Black, Indigenous, and children of color; whereby Black and Hispanic populations have been shown to experience greater adverse mental health outcomes associated with temperature, including anxiety, psychosis, and substance use disorders, compared to white populations [88]. Research has also shown that housing type, like multifamily rental units, and housing availability with air conditioning are important determinants of heat risk disparities, particularly for urban, non-White communities [89, 90]. Results showed that broadband access was an important predictor for high-risk clustering of suicide during compounding events. Digital exclusion or more broadly limited Internet connectivity may be a significant modifiable risk factor to enhance access to extreme weather warnings and mental health support resources for rural, non-English speaking, and low-income or BIPOC families.

Greenspace has been linked with environmental health benefits including improved air quality and cooling and shade from extreme heat [91–93]. Several studies have demonstrated the protective mental health effects of urban greenspace, including a reduction in depression and suicide [94]. For children, green or park space provides a safe space to play and connect with others. Research has shown a strong linkage between the greenest neighborhoods and fewer psychiatric conditions in this age group, as well as improved mental health and well-being and neurocognitive development [95–97]. A recent longitudinal study observed a strong positive relationship between higher average temperatures and aggressive behaviors in children and revealed that children who resided in neighborhoods with high compared to low concentrations of greenspace experienced a reduction in aggressive behavior [28]. More policy-focused and health intervention research around the co-benefits of increased access to greenspace on health and climate change adaptation and mitigation is needed.

### Strengths and limitations

Prior studies have revealed significant spatial clustering of mortality in older adults during heatwaves in Paris [98] and increased ED visits during heatwaves in rural communities [99]. None have examined the spatial co-occurrence of multiple climate hazards and related health impacts on child mental health. By highlighting at-risk locations and this sensitive population, spatial analysis improved limited understanding on the effects of co-occurring climate hazards. Exploratory findings from this study can be used to generate and test causal hypotheses in future research on the interlinkages between social determinants and physical climate hazards to identify factors that can be leveraged to enhance adaptive actions (e.g., increase availability of greenspace and broadband access) in at-risk communities. In addition, this study utilized data from the community resilience equity supplement, a product that integrates publicly unavailable microdata from the Census, American Community Survey, and Planning database to provide more precise yearly estimates of community resilience at the county scale [60]. CRE estimates are an enhancement from the typical ACS 1-year data because the data used to derive these estimates originate from microdata from multiple data sources, including the 2010 Decennial Census, Population Estimates Program of the U.S. Census Bureau, and internal ACS microdata to produce ACS estimates from various geographies. Lastly, this study relied on the robust MARS model, a machine learning algorithm that, unlike other variable selection techniques, makes no assumptions between the independent predictors. Each predictor is scanned by the algorithm to identify splits that improve accuracy and unlike ordinary least squares regression models, correlation among variables does not impede predictive accuracy [73, 74].

Our results are limited due to the retrospective and ecological study design. Ecological designs can introduce the modifiable areal unit problems (MAUPs), as data aggregation can introduce statistical bias increasing the magnitude of the effect at larger spatial scales (e.g., county) relative to smaller scales (e.g., individual household, zip code). Similarly, because the administrative health data and drought data were only available at the county scale, we characterized exposure to physical climate hazards by relying on data aggregated to the county boundary. This analysis considered only one form of multi-hazard events, those with compound temporal and spatial relationships. While other forms of these relationships exist such as consecutive, where two or more hazards occur in succession and their direct impacts overlap spatially before recovery from a previous event is complete [100], may exhibit differential mental health relationships/outcomes across compounding extreme hazard types. Lastly, while we relied on clinician diagnosis recorded in hospital administrative data, a common epidemiologic approach, we did not have personal exposure data for each ED visit that linked mood or suicidal tendencies with weather conditions for that day. Future analyses are needed at different scales (e.g., the census tract) to better characterize both mental health and ambient exposure to physical hazards.

## Conclusion

There has been no research to-date on the concurrent impact of both heatwave and drought events on child mental health burden. Our findings revealed clusters of high suicide and mood disorder risks for pediatric populations during heatwaves, drought, and compounding event days. Important predictive factors included the availability of residential green or park space, housing availability, residential segregation, vehicle access, and low English proficiency. There is a growing call for a more reparative approach to advance community resilience that includes not just a wealth transfer to address the legacies of injustice, but also a shift towards more equitable climate change policymaking. Examples might include granting reparations and advancing land reclamations, scoring equity outcomes in climate change policy, enhancing wealth and financial security of low-income households, investing in localized climate adaptation in majority-Black neighborhoods, and integrating health into climate change policy [101]. Public health intervention efforts focused on mental health risk reduction might include local policies that increase transportation and broadband access, more targeted communication efforts in Latinx communities, and low-cost mental health support (e.g., crisis text or phone hotlines) during these compounding extreme weather events.

### Supplementary Information

Below is the link to the electronic supplementary material.

Supplementary file1 (DOCX 27 KB)

## Data Availability

The datasets analyzed during the current study are not publicly available because they contain protected health information and are not shareable via stipulations in the data use agreement.

## References

[1] Seneviratne SI, Zhang X, Adnan M, Badi W, Dereczynski C, Luca AD, Zhou B (2021). Chapter 11: weather and climate extreme events in a changing climate. Climate change 2021: The Physical Science Basis Contribution of Working Group I to the Sixth Assessment Report of the Intergovernmental Panel on Climate Change.

[2] Leonard M, Westra S, Phatak A, Lambert M, Hurk B, Mcinnes K, Stafford Smith M (2014). A compound event framework for understanding extreme impacts. Wiley Interdiscip Rev Clim Change.

[3] Zscheischler J, Westra S, van den Hurk BJJM, Seneviratne SI, Ward PJ, Pitman A, Zhang X (2018). Future climate risk from compound events. Nat Clim Change.

[4] Stott PA, Stone DA, Allen MR (2004). Human contribution to the European heatwave of 2003. Nature.

[5] Ionita M, Tallaksen LM, Kingston DG, Stagge JH, Laaha G, Van Lanen HAJ, Haslinger K (2017). The European 2015 drought from a climatological perspective. Hydrol Earth Syst Sci.

[6] Grumm RH (2011). The Central European and Russian heat event of July–August 2010. Bull Am Meteor Soc.

[7] Turco M, von Hardenberg J, AghaKouchak A, Llasat MC, Provenzale A, Trigo RM (2017). On the key role of droughts in the dynamics of summer fires in Mediterranean Europe. Sci Rep.

[8] Fink AH, Brücher T, Krüger A, Leckebusch GC, Pinto JG, Ulbrich U (2004). The 2003 European summer heatwaves and drought –synoptic diagnosis and impacts. Weather.

[9] Zampieri M, Ceglar A, Dentener F, Toreti A (2017). Wheat yield loss attributable to heat waves, drought and water excess at the global, national and subnational scales. Environ Res Lett.

[10] Lu J, Metzger K, Cajigal A, Konty K, Matte T (2007). Identifying and modeling spatial patterns of heat-related illness in New York City. Adv Dis Surveill.

[11] EC. Addressing the challenge of water scarcity and droughts in the European Union—European Environment Agency. Eurpoean Environment Agency. 2007. https://www.eea.europa.eu/policy-documents/addressing-the-challenge-of-water. Accessed 11 June 2023.

[12] Sutanto SJ, Vitolo C, Di Napoli C, D’Andrea M, Van Lanen HAJ (2020). Heatwaves, droughts, and fires: exploring compound and cascading dry hazards at the pan-European scale. Environ Int.

[13] AghaKouchak A, Cheng L, Mazdiyasni O, Farahmand A (2014). Global warming and changes in risk of concurrent climate extremes: insights from the 2014 California drought. Geophys Res Lett.

[14] Steffen W, Mallon K, Kompas T, Dean A, Rice M. Compound costs: how climate change is damaging Australia’s economy (Report). Climate Council. 2019. https://apo.org.au/node/234731. Accessed 5 May 2023.

[15] Zscheischler J, Seneviratne SI (2017). Dependence of drivers affects risks associated with compound events. Sci Adv.

[16] Herrera-Estrada JE, Sheffield J (2017). Uncertainties in future projections of summer droughts and heat waves over the contiguous United States. J Clim.

[17] Sarhadi A, Ausín MC, Wiper MP, Touma D, Diffenbaugh NS (2018). Multidimensional risk in a nonstationary climate: Joint probability of increasingly severe warm and dry conditions. Sci Adv.

[18] Hao Z, Hao F, Singh VP, Zhang X (2018). Changes in the severity of compound drought and hot extremes over global land areas. Environ Res Lett.

[19] Alizadeh MR, Adamowski J, Nikoo MR, AghaKouchak A, Dennison P, Sadegh M (2020). A century of observations reveals increasing likelihood of continental-scale compound dry-hot extremes. Sci Adv.

[20] Wu X, Hao Z, Tang Q, Singh VP, Zhang X, Hao F (2021). Projected increase in compound dry and hot events over global land areas. Int J Climatol.

[21] Copernicus Climate Change Service (C3S). *Copernicus Climate Change Service (C3S), 2023: European State of the Climate 2022* (No. 6). 2023. https://climate.copernicus.eu/extreme-heat-widespread-drought-typify-european-climate-2022. Accessed 5 May 2023.

[22] Helldén D, Andersson C, Nilsson M, Ebi KL, Friberg P, Alfvén T (2021). Climate change and child health: a scoping review and an expanded conceptual framework. Lancet Planet Health.

[23] Vergunst F, Berry HL (2022). Climate change and children’s mental health: a developmental perspective. Clin Psychol Sci.

[24] Mazdiyasni O, AghaKouchak A (2015). Substantial increase in concurrent droughts and heatwaves in the United States. Proc Natl Acad Sci.

[25] Perera F, Nadeau K (2022). Climate change, fossil-fuel pollution, and children’s health. N Engl J Med.

[26] Thiery W, Lange S, Rogelj J, Schleussner C-F, Gudmundsson L, Seneviratne SI, Wada Y (2021). Intergenerational inequities in exposure to climate extremes. Science.

[27] Basu R, Gavin L, Pearson D, Ebisu K, Malig B (2017). Examining the association between apparent temperature and mental health-related emergency room visits in California. Am J Epidemiol.

[28] Younan D, Li L, Tuvblad C, Wu J, Lurmann F, Franklin M, Chen J-C (2018). Long-term ambient temperature and externalizing behaviors in adolescents. Am J Epidemiol.

[29] Dean JG, Stain HJ (2010). Mental health impact for adolescents living with prolonged drought. Aust J Rural Health.

[30] Wang X, Lavigne E, Ouellette-kuntz H, Chen BE (2014). Acute impacts of extreme temperature exposure on emergency room admissions related to mental and behavior disorders in Toronto, Canada. J Affect Disord.

[31] Page LA, Hajat S, Kovats RS (2007). Relationship between daily suicide counts and temperature in England and Wales. Br J Psychiatry.

[32] Hansen A, Bi P, Nitschke M, Ryan P, Pisaniello D, Tucker G (2008). The effect of heat waves on mental health in a temperate Australian city. Environ Health Perspect.

[33] Khalaj B, Lloyd G, Sheppeard V, Dear K (2010). The health impacts of heat waves in five regions of New South Wales, Australia: a case-only analysis. Int Arch Occup Environ Health.

[34] Ding N, Berry H, O’Brien L (2015). The effect of extreme heat on mental health—evidence from Australia. Int J Epidemiol.

[35] Nori-Sarma A, Sun S, Sun Y, Spangler KR, Oblath R, Galea S, Wellenius GA (2022). Association between ambient heat and risk of emergency Department visits for mental health among US adults, 2010 to 2019. JAMA Psychiatry.

[36] Barkin JL, Buoli M, Curry CL, von Esenwein SA, Upadhyay S, Kearney MB, Mach K (2021). Effects of extreme weather events on child mood and behavior. Dev Med Child Neurol.

[37] Li M, Gu S, Bi P, Yang J, Liu Q (2015). Heat waves and morbidity: current knowledge and further direction-a comprehensive literature review. Int J Environ Res Public Health.

[38] Knowlton K, Rotkin-Ellman M, King G, Margolis HG, Smith D, Solomon G, English P (2009). The 2006 California heat wave: impacts on hospitalizations and emergency department visits. Environ Health Perspect.

[39] Xu Z, Sheffield PE, Su H, Wang X, Bi Y, Tong S (2014). The impact of heat waves on children’s health: a systematic review. Int J Biometeorol.

[40] Lõhmus M (2018). Possible biological mechanisms linking mental health and heat—a contemplative review. Int J Environ Res Public Health.

[41] Stanke C, Kerac M, Prudhomme C, Medlock J, Murray V (2013). Health effects of drought: a systematic review of the evidence. PLoS Curr.

[42] Tripathy KP, Mukherjee S, Mishra AK, Mann ME, Williams AP (2023). Climate change will accelerate the high-end risk of compound drought and heatwave events. Proc Natl Acad Sci.

[43] Keellings D, Engström J (2019). The future of drought in the Southeastern U.S.: projections from downscaled CMIP5 models. Water.

[44] Berg A, Lintner BR, Findell K, Seneviratne SI, van den Hurk B, Ducharne A, Gentine P (2015). Interannual coupling between summertime surface temperature and precipitation over land: processes and implications for climate change. J Clim.

[45] Durre I, Arguez A, Schreck CJ, Squires MF, Vose RS (2022). Daily high-resolution temperature and precipitation fields for the contiguous United States from 1951 to present. J Atmos Oceanic Tech.

[46] Anderson BG, Bell ML (2009). Weather-related mortality: how heat, cold, and heat waves affect mortality in the United States. Epidemiology.

[47] Lyon B (2009). Southern Africa summer drought and heat waves: observations and coupled model behavior. J Clim.

[48] Grundstein A, Dowd J (2011). Trends in extreme apparent temperatures over the United States, 1949–2010. J Appl Meteorol Climatol.

[49] Cowan T, Hegerl GC, Colfescu I, Bollasina M, Purich A, Boschat G (2017). Factors contributing to record-breaking heat waves over the great plains during the 1930s dust bowl. J Clim.

[50] Shafiei Shiva J, Chandler DG, Kunkel KE (2019). Localized changes in heat wave properties across the United States. Earth’s Future.

[51] Rennie J, Bell JE, Kunkel KE, Herring S, Cullen H, Abadi AM (2019). Development of a submonthly temperature product to monitor near-real-time climate conditions and assess long-term heat events in the United States. J Appl Meteorol Climatol.

[52] Leeper RD, Bell JE, Palecki MA (2019). A description and evaluation of U.S. climate reference network standardized soil moisture dataset. J Appl Meteorol Climatol..

[53] Svoboda M, LeComte D, Hayes M, Heim R, Gleason K, Angel J, Stephens S (2002). The drought monitor. Bull Am Meteorol Soc.

[54] Leeper RD, Bilotta R, Petersen B, Stiles CJ, Heim R, Fuchs B, Prat OP, Palecki M, Ansari S (2022). Characterizing U.S. drought over the past 20 years using the U.S. drought monitor. Int J Climatol.

[55] Massey DS, Fischer MJ (2000). How segregation concentrates poverty. Ethn Racial Stud.

[56] Massey DS (1996). The age of extremes: concentrated affluence and poverty in the Twenty-First Century. Demography.

[57] Krieger N, Singh N, Waterman PD (2016). Metrics for monitoring cancer inequities: residential segregation, the Index of Concentration at the Extremes (ICE), and breast cancer estrogen receptor status (USA, 1992–2012). Cancer Causes Control.

[58] Hardeman RR, Homan PA, Chantarat T, Davis BA, Brown TH (2022). Improving the measurement of structural racism to achieve antiracist health policy. Health Aff.

[59] Handwerger LR, Sugg MM, Runkle JD (2021). Present and future sea level rise at the intersection of race and poverty in the Carolinas: a geospatial analysis. J Clim Change Health.

[60] U.S. Census Bureau. 2019 Community Resilience Estimates Equity Supplement File Layout. United States Census Bureau. (2021, September 21). https://www2.census.gov/programs-surveys/demo/technical-documentation/community-resilience/2019/cre_for_equity_file_layout.pdf. Accessed 25 July 2023.

[61] U.S. Geological Survey (USGS) Gap Analysis Project (GAP). (2022). Protected Areas Database of the United States (PAD-US) 3.0 (ver. 2.0, March 2023). U.S. Geological Survey. 10.5066/P9Q9LQ4B

[62] The Trust for Public Land. ParkServe® Data Downloads. The Trust for Public Land. 2023. https://www.tpl.org/park-data-downloads. Accessed 25 July 2023.

[63] Runkle JD, Matthews JL, Sparks L, McNicholas L, Sugg MM. Racial and ethnic disparities in pregnancy complications and the protective role of greenspace: a retrospective birth cohort study. Sci Total Environ. 2022;808. 10.1016/j.scitotenv.2021.152145. 10.1016/j.scitotenv.2021.152145 34871679

[64] Sprague NL, Bancalari P, Karim W, Siddiq S (2022). Growing up green: a systematic review of the influence of greenspace on youth development and health outcomes. J Eposure Sci Environ Epidemiol.

[65] Kulldorff M, Huang L, Pickle L, Duczmal L (2006). An elliptic spatial scan statistic. Stat Med.

[66] Talbot T, Kumar S, Kulldorff M. The Bernoulli Spatial Scan Statistic for Birth Defect Data. 2015. https://www.satscan.org/tutorials/nysbirthdefect/SaTScanTutorialNYSBirthDefect.pdf. Accessed 5 May 2023.

[67] Kulldorff M. SaTScan User Guide for version 10.1. 2022, July 1. https://www.satscan.org/cgi-bin/satscan/register.pl/SaTScan_Users_Guide.pdf?todo=process_userguide_download.

[68] Tango T (2021). Spatial scan statistics can be dangerous. Stat Methods Med Res.

[69] VanderWeele TJ, Knol MJ (2014). A tutorial on interaction. Epidemiol Methods.

[70] Greenland S (2009). Interactions in epidemiology: relevance, identification, and estimation. Epidemiology (Cambridge, Mass.).

[71] Rothman KJ, Greenland S (2005). Causation and causal inference in epidemiology. Am J Public Health.

[72] Knol MJ, VanderWeele TJ, Groenwold RHH, Klungel OH, Rovers MM, Grobbee DE (2011). Estimating measures of interaction on an additive scale for preventive exposures. Eur J Epidemiol.

[73] Buja A, Duffy D, Hastie T, Tibshirani R (1991). Discussion: multivariate adaptive regression splines. Ann Stat.

[74] Hastie T, Tibshirani R, Friedman J (2009). The elements of statistical learning.

[75] Friedman JH, Silverman BW (1989). Flexible parsimonious smoothing and additive modeling. Technometrics.

[76] Friedman JH. Estimating functions of mixed ordinal and categorical variables using adaptive splines. 1991. https://apps.dtic.mil/sti/citations/ADA590939. Accessed 6 May 2023.

[77] Kuhfeld W, Cai W. 457–2013: Introducing the New ADAPTIVEREG Procedure for Adaptive Regression. *SAS Global Forum 2013: Statistics and Data Analysis*. 2013. http://support.sas.com/resources/papers/proceedings13/457-2013.pdf. Accessed 9 May 2023.

[78] Knafl GJ, Ding K (2016). Adaptive regression for modeling nonlinear relationships.

[79] SAS. (n.d.). SAS/STAT 13.1 User’s guide documentation examples. SAS analytics and software & solutions. http://support.sas.com/documentation/onlinedoc/stat/examples/131/index.html. Accessed 5 May 2023.

[80] Becker-Blease KA, Turner HA, Finkelhor D (2010). Disasters, victimization, and children’s mental health. Child Dev.

[81] Basu S, Banerjee B (2020). Impact of environmental factors on mental health of children and adolescents: a systematic review. Child Youth Serv Rev.

[82] Carnie T-L, Berry HL, Blinkhorn SA, Hart CR (2011). In their own words: young people’s mental health in drought-affected rural and remote NSW. Aust J Rural Health.

[83] Nicholls N, Butler CD, Hanigan I (2006). Inter-annual rainfall variations and suicide in New South Wales, Australia, 1964–2001. Int J Biometeorol.

[84] OBrien LV, Berry HL, Coleman C, Hanigan IC (2014). Drought as a mental health exposure. Environ Res.

[85] Burke M, González F, Baylis P, Heft-Neal S, Baysan C, Basu S, Hsiang S (2018). Higher temperatures increase suicide rates in the United States and Mexico. Nat Clim Chang.

[86] Thompson R, Hornigold R, Page L, Waite T (2018). Associations between high ambient temperatures and heat waves with mental health outcomes: a systematic review. Public Health.

[87] Bernstein AS, Sun S, Weinberger KR, Spangler KR, Sheffield PE, Wellenius GA (2022). Warm season and emergency department visits to U.S. Children’s hospitals. Environ Health Perspect.

[88] Berberian AG, Gonzalez DJX, Cushing LJ (2022). Racial disparities in climate change-related health effects in the United States. Curr Environ Health Rep.

[89] Uejio CK, Wilhelmi OV, Golden JS, Mills DM, Gulino SP, Samenow JP (2011). Intra-urban societal vulnerability to extreme heat: the role of heat exposure and the built environment, socioeconomics, and neighborhood stability. Health Place.

[90] Gabbe CJ, Mallen E, Varni A (2022). Housing and urban heat: assessing risk disparities. Housing Policy Debate.

[91] Bowler DE, Buyung-Ali L, Knight TM, Pullin AS (2010). Urban greening to cool towns and cities: a systematic review of the empirical evidence. Landsc Urban Plan.

[92] Depietri Y, Renaud F, Kallis G (2012). Heat waves and floods in urban areas: a policy-oriented review of ecosystem services. Sustain Sci.

[93] Nowak DJ, Hirabayashi S, Bodine A, Greenfield E (2014). Tree and forest effects on air quality and human health in the United States. Environ Pollut.

[94] Braubach M, Egorov A, Mudu P, Wolf T, Ward Thompson C, Martuzzi M, Kabisch N, Korn H, Stadler J, Bonn A (2017). Effects of urban green space on environmental health, equity and resilience. Nature-based solutions to climate change adaptation in urban areas: linkages between science, policy and practice.

[95] Maas J, Verheij RA, de Vries S, Spreeuwenberg P, Schellevis FG, Groenewegen PP (2009). Morbidity is related to a green living environment. J Epidemiol Community Health.

[96] McCormick R (2017). Does access to green space impact the mental well-being of children: a systematic review. J Pediatr Nurs Nurs Care Child Fam.

[97] Vanaken G-J, Danckaerts M (2018). Impact of green space exposure on children’s and adolescents’ mental health: a systematic review. Int J Environ Res Public Health.

[98] Benmarhnia T, Kihal-Talantikite W, Ragettli MS, Deguen S (2017). Small-area spatiotemporal analysis of heatwave impacts on elderly mortality in Paris: a cluster analysis approach. Sci Total Environ.

[99] Bishop-Williams KE, Berke O, Pearl DL, Kelton DF (2015). A spatial analysis of heat stress related emergency room visits in rural Southern Ontario during heat waves. BMC Emerg Med.

[100] de Ruiter MC, Couasnon A, van den Homberg MJC, Daniell JE, Gill JC, Ward PJ (2020). Why we can no longer ignore consecutive disasters. Earth’s Future.

[101] Donoghoe M, Perry AM. The case for climate reparations in the United States | Brookings. Brookings. (2023, March 1). https://www.brookings.edu/articles/the-case-for-climate-reparations-in-the-united-states/. Accessed 24 July 2023.

