# Supplementary material for: Impacts of compounding drought and heatwave events on child mental health: insights from a spatial clustering analysis
Source: Discov Ment Health. 2024 Jan 2;4(1):1. doi: 10.1007/s44192-023-00055-0 (PMC10761644; doi:10.1007/s44192-023-00055-0)
Supplement: Supplementary file 1 — Supplementary file1 (DOCX 27 KB) [file 44192_2023_55_MOESM1_ESM.docx]

**Supplemental Materials**

**Supplemental Table 1.** Variables from the CRE for Equity included in the analysis known to influence vulnerability, exposure and adaptive responses to climate hazards

| **Variable** | **Description** |
| --- | --- |
| crowd_occ_PE | Percentage of occupied housing units with 1.51 occupants or more per room |
| civ_vet_PE | Percentage of the population 18 years and over that are civilian veterans |
| Rural_Pop_PE | Percentage of the population living in a rural area during 2010 Census. |
| Rent_Vac_PE | Rental vacancy rate |
| No_Veh_PE | Percentage of occupied housing units with no vehicles available. |
| No_Health_Ins_PE | Percentage of civilian noninstitutionalized population with no health insurance coverage. |
| Median_age_E | Median age of the population. |
| Housing_Units_E | Estimated number of housing units. |
| Hispanic_PE | Hispanic or Latino population (%) |
| HS_Grad_PE | Percentage of population 25 and over that are a high school graduate or higher |
| HO_Vac_PE | Homeowner vacancy rate |
| Female_no_partner_w_child_PE | Percentage of households with a female householder, no spouse/partner present, with own children of household under 18 years |
| 65_plus_PE | Percentage of the population 65 years and over |
| ENG_LVW_PE | Percentage of the population 5 years and over that speaks a language other than English and speak English less than "very well". |
| Disabled_PE | Percentage of civilian noninstitutionalized population with a disability. |
| Broadband_PE | Percentage of households with a broadband Internet subscription |

**Supplemental Table 2.** Summary statistics for average ambient temperature for heatwaves and heatwaves during a drought in the warm (May-Sept_ season across all 100 counties in NC, 2016-2019.

|  | **Heatwave** | **Heatwaves during droughts** | **High-intensity Heatwaves** | **High-intensity Heatwaves during droughts** |
| --- | --- | --- | --- | --- |
| **N (Days)** | 1,060 | 81 | 2,321 | 815 |
| **Tavg Mean °C (SD)** | 33.2 (2.5) | 32.3 (3.4) | 33.0 (2.5) | 31.3 (2.7) |

**Supplemental Table 3.**  Adaptive regression output for sensitivity analysis using high intensity heatwave definition for (A) Mood Disorders and (B) Suicidality.

| 1. **Mood Disorders** | | | | | | | | | | | |  |
| --- | --- | --- | --- | --- | --- | --- | --- | --- | --- | --- | --- | --- |
| *Residence in high-risk cluster during high intensity heatwave* | | | *Residence in high-risk cluster during compounding drought and*  *high intensity heatwave* | | | | | |  |  |  |  |
| Variable | Bases | Importance |  | | | | Bases | Importance |  |  |  |  |
| % of population 25 years + that are a high school | 4 | 100 | % of occupied housing units with 1.51 occupants/room | | | | 3 | 100 |  |  |  |  |
| homeowner vacancy rate | 2 | 94.50 | % of households with broadband internet | | | | 4 | 98.44 |  |  |  |  |
| % of occupied housing units with 1.51 occupants/room | 2 | 62.75 | % of occupied housing units with no vehicles available | | | | 2 | 95.43 |  |  |  |  |
| % of population 18 years + that are civilian veterans | 3 | 54.10 | % of population 25 years + that are HS graduates or higher | | | | 3 | 84.04 |  |  |  |  |
| % of population 65 years+ | 2 | 53.27 | number of housing units | | | | 2 | 56.38 |  |  |  |  |
| residential segregation | 2 | 48.54 | % total greenspace per person | | | | 2 | 34.68 |  |  |  |  |
|  |  |  | % of population Hispanic or Latino | | | | 4 | 34.27 |  |  |  |  |
|  |  |  | Residential segregation | | | | 2 | 28.53 |  |  |  |  |
| *Model Fit Statistics* | | | | | | | | | | | |  |
| GCV |  | 0.07055 |  |  |  |  | 0.06588 | | | | | |
| GCV R-Square |  | 0.90306 |  |  |  |  | 0.89402 | | | | | |
| Effective Degrees of Freedom |  | 31 |  |  |  |  | 47 | | | | | |
| Log Likelihood |  | -948.5708 |  |  |  |  | -270.4325 | | | | | |
| Deviance |  | 141.1415 |  |  |  |  | 22.8651 | | | | | |
|  |  |  |  | | | |  |  |  |  |  |  |
|  |  |  |  | | | |  |  |  |  |  |  |
|  |  |  |  | | | |  |  |  |  |  |  |
|  |  |  |  | | | |  |  |  |  |  |  |
| 1. **Suicidality** | | | | | | | | | | | |  |
| *Residence in high-risk cluster during high intensity heatwave* | | | *Residence in high-risk cluster during compounding*  *drought and high intensity heatwave* | | | | | |  |  |  |  |
| Variable | Bases | Importance |  | | | | Bases | Importance |  |  |  |  |
| residential segregation | 4 | 100 | % of population 25 years + that are a high school | | | | 3 | 100 |  |  |  |  |
| Rental vacancy rate | 3 | 84.86 | residential segregation | | | | 2 | 79.02 |  |  |  |  |
| % total greenspace per person | 2 | 84.86 | % of civilian non-institutionalized population with no health insurance | | | | 4 | 75.98 |  |  |  |  |
| estimated number of housing units | 2 | 65.27 | homeowner vacancy rate | | | | 2 | 50.52 |  |  |  |  |
| % of population Hispanic or Latino | 2 | 64.69 | % of population 65 years+ | | | | 2 | 43.05 |  |  |  |  |
|  |  |  | % of occupied housing units with no vehicles available | | | | 2 | 39.92 |  |  |  |  |
|  |  |  |  | | | |  |  |  |  |  |  |
| *Model Fit Statistics* | | | | | | | | | | | |  |
| GCV |  | 0.0617 |  | | | |  | 0.02579 |  |  |  |  |
| GCV R-Square |  | 0.9878 |  | | | |  | 0.9966 |  |  |  |  |
| Effective Degrees of Freedom |  | 29 |  | | | |  | 31 |  |  |  |  |
| Log Likelihood |  | -264.00 |  | | | |  | -62.00 |  |  |  |  |
| Deviance |  | 0.0615 |  | | | |  | 0.0510 |  |  |  |  |
